# Brain Gray Matter Abnormalities in First-Episode, Treatment-Naive Children with Obsessive-Compulsive Disorder

**DOI:** 10.3389/fnbeh.2016.00141

**Published:** 2016-06-30

**Authors:** Bochao Cheng, Wu Cai, Xiuli Wang, Du Lei, Yingkun Guo, Xun Yang, Qizhu Wu, Jianping Gong, Qiyong Gong, Gang Ning

**Affiliations:** ^1^Department of Radiology, West China Second University Hospital of Sichuan UniversityChengdu, China; ^2^Department of Radiology, Huaxi MR Research Center, West China Hospital of Sichuan UniversityChengdu, China; ^3^Department of Radiology, The Second Affiliated Hospital of Soochow UniversitySuzhou, China; ^4^Department of Psychosis Studies, Institute of Psychiatry, Psychology and Neuroscience, King’s College LondonLondon, UK; ^5^School of Sociality and Psychology, Southwest University for NationalitiesChengdu, China; ^6^Monash Biomedical Imaging, Monash UniversityClayton, VIC, Australia

**Keywords:** obsessive compulsive disorder, pediatrics, treatment-naive, voxel-based morphometry, gray matter volume

## Abstract

Although several magnetic resonance imaging (MRI) studies have been conducted in children with obsessive-compulsive disorder (OCD), the brain structural abnormalities in OCD, especially in children, are not yet well characterized. We aimed to identify gray matter (GM) abnormalities in the early stage of pediatric OCD and examine the relationship between these structural abnormalities with clinical characteristics. Examinations of 30 first-episode, treatment-naive pediatric OCD patients without any comorbidities and 30 matched healthy controls (HCs) were performed with 3.0 T magnetic resonance imaging (MRI). Voxel-based morphometry (VBM) following Diffeomorphic Anatomical Registration using Exponentiated Lie algebra (DARTEL) was used to conduct voxel-wise tests for group differences in regional gray matter volume (GMV). Compared to HCs, the patient group exhibited more GMV in the bilateral putamen and left orbitofrontal cortex (OFC) and less GMV in the left inferior parietal lobule (IPL). The GMV alternation in the right putamen of OCD patients was positively correlated with Hamilton Anxiety Rating Scale (HAM-A) scores, while the GMV alternation in the left IPL exhibited a trend to negatively correlate with HAM-A scores. Our current results suggest that the GM abnormalities were defined in the early stage of pediatric OCD. Moreover, these findings provided further evidence of brain GM abnormalities that are not only present in the classical fronto–striatal–thalamic circuit but also in the default mode network (DMN), which may represent the interaction of abnormally functional organization of both network in pediatric OCD.

## Introduction

Obsessive–compulsive disorder (OCD) is a common and clinically heterogeneous mental disorder that has a lifetime prevalence rate of 2–3% in the general population (Rasmussen and Eisen, 1992). OCD is characterized by the presence of recurrent, persistent, unreasonable thoughts (obsessions) and repetitive behaviors (compulsions; American Psychiatric Association, 2000). According to the new Diagnostic and Statistical Manual of Mental Disorders 5 (DSM-5) system, OCD is no longer included in the anxiety disorder category and is instead included under the umbrella of “obsessive-compulsive and related disorders”.

Current neuroimaging tools have the potential to elucidate the neurobiological bases of psychiatric disorders. The abnormal feedback loops within the fronto–striatal–thalamic circuithave been described in the symptoms and pathophysiology of OCD (Eng et al., 2015). Particularly, the dysfunction of the frontal structures (including the orbitofrontal cortex, OFC), basal ganglia and anterior cingulate cortex (ACC) were reported to account for the principal features of OCD by functional magnetic resonance imaging (fMRI) studies (Rauch et al., 1994; Graybiel and Rauch, 2000; Eng et al., 2015). These studies reported considerably unified results in adult OCD, both in a resting-state (Baxter et al., 1988) and during task performance (Rauch et al., 1994).

Although several structural MRI studies (Rosenberg et al., 1997; de Wit et al., 2014), including meta-analyses (Rotge et al., 2010; Piras et al., 2015), of adult OCD have revealed structural alternation in the fronto–striatal–thalamic circuit, the results are heterogeneous. For example, Aylward et al. (1996) failed to identify any cerebral structural abnormalities in adult OCD. Pujol et al. (2004) reported less gray matter volume (GMV) in the medial frontal gyrus and OFC but more GMV in the bilateral putamen and the cerebellumin adult OCD. Valente et al. (2005) identified more GMV in the OFC and parahippocampal regions as well as less ACC in adult OCD. These divergences may partly stem from the heterogeneity of the samples enrolled and the medications used. In addition, the parietal and temporal cortices were also reported to be involved in OCD in structural MRI studies (Menzies et al., 2008).

Remarkably, OCD patients vary widely with respect to symptoms, age of illness onset, disease duration and comorbidities (Brady, 2014). Both selective serotonin reuptake inhibitors (SSRIs) and cognitive-behavioral therapy in OCD have also been linked to gray matter (GM) alternation (Szeszko et al., 2004; Huyser et al., 2012). In addition, prior studies revealed that the adult and pediatric OCD have different cerebral structural alternations in the same anatomical regions (Rosenberg et al., 1997; Pujol et al., 2004). Because as many as 80% of adult OCD patients exhibit a pediatric onset (Yousefi Chaijan et al., 2014), first-episode, treatment-naive pediatric OCD patients provide the opportunity to investigate OCD at an early stage without confounding factors such as the effect of disease duration, treatment and the disorder itself on brain development.

The cerebral abnormalities of pediatric OCD have been reported in previous structural MRI studies (Szeszko et al., 2004; 2008; Carmona et al., 2007; Lázaro et al., 2009, 2014). Most of these studies were based on volumetric region-of-interest (ROI) methods (Szeszko et al., 2004), and few studies utilized voxel-based morphometry (VBM). Notably, some voxel-based MRI studies in pediatric OCD (Gilbert et al., 2008; Lázaro et al., 2009, 2014) utilized VBM with the SPM adult brain template or create a study-specific template (Carmona et al., 2007). To improve inter-subjects registration of the MRI images (Ashburner, 2007), Diffeomorphic Anatomic Registration Through Exponentiated Lie algebra algorithm (DARTEL) was used to create individual templates, which had been applied to evaluate within-subject changes in previous pediatric OCD studies (Huyser et al., 2012). Moreover, Klein et al. (2009) carried out a largest evaluation of nonlinear deormation algorithms applied to brain image registration, which included four different VBM algorithms (e.g., DARTEL). DARTEL was found to rank the top level to optimize the sensitivity of such analysis and detect subtle brain structural changes than those measured with standard VBM.

In the current study, we aimed to identify GM abnormalities with VBM-DARTEL in the early stage of pediatric OCD and examine the relationship between these structural abnormalities with clinical characteristics. We strictly enrolled first-episode, treatment-naive pediatric OCD patients without any comorbidities to exclude their potential effects on the observed findings. We hypothesized that pediatric OCD patients have GM abnormalities in the early stage and that these abnormalities are present not only in the cortical-striatal-thalamic circuit but also in other brain regions, such as the parietal and/or temporal cortices. Additionally, we expect to find relationships between the GM structural abnormalities and clinical characteristics.

## Materials and Methods

### Study Design and Participants

The Ethics Committee of the Second University Hospital of Soochow University approved the current study. The age range of all participants was 8–14 years (Mean = 10.6, SD = 2.2). All participants were right-handed with a full-scale intelligence quotient (IQ) above 70, according to the Wechsler Intelligence Scale-fifth edition for Children (WISC-V; Wechsler, 2014). The study was conducted in accordance with the ethical principles from the Declaration of Helsinki, consistent Good Clinical Practices, and applicable regulatory requirements. Written informed consent was obtained from all participants and their parents.

Thirty consecutive children with OCD were assessed by two experienced child psychiatrists (XKP and XH) at the Departments of Neurology and Psychiatry of the Second University Hospital of Soochow University. Patients were diagnosed using the DSM-IV (American Psychiatric Association, 2000) criteria and the Schedule for Affective Disorders and Schizophrenia for School Age Children—Present and Lifetime (KSADS-PL; Kaufman et al., 1997). All patients were experiencing their first episode of OCD and were treatment-naive without any comorbidities, such as developmental disorders, schizophrenia and other psychotic disorders, mood disorders, disruptive behavior disorders and eating disorders. The exclusion criteria included an IQ below 70, the use of psychotropic medication or CBT, a personal history of neurological and psychiatric disorders, a history of head injury, alcohol or drug abuse, mental retardation Pediatric Autoimmune Neuropsychiatric Disorders Associated with Streptococcus (PANDAS) or Pediatric Acute-Onset Neuropsychiatric Syndrome (PANS) and all other DSM-IV Axis I disorders.

Thirty healthy controls (HCs) were recruited from local schools via poster advertisements. HCs were matched for age, gender, IQ and education. Exclusion criteria included a personal history of neurological illness and psychiatric disorders and the other exclusion criteria mentioned in the patient group.

To assess the severity of OCD symptoms, the Children’s Yale-Brown Obsessive–Compulsive Scale (CY-BOCS; Scahill et al., 1997) was used (above score 16). The CY–BOCS assesses the severity of both obsessions and compulsions separately and provides an overall score (Scahill et al., 1997). In addition, we used the 17-item Hamilton Rating Scale for Depression (HAM-D; Hamilton, 1967) and the Hamilton Rating Scale for Anxiety (HAM-A; Hamilton, 1959) to rate the severity of participants’ depressive and anxiety symptoms prior to their MRI scans.

### MRI Acquisition

Brain MR images were obtained on a 3.0 T MRI system equipped with a 16 channel head coil (Achieva; Philips Medical Systems, Best, Netherlands). Each participant underwent a high-resolution 3D T1­weighted structural scan with T1WI_3D_TFE sequence. The imaging parameters were as follows: voxel size = 1 × 1.25 × 2 mm, 1 mm thick adjacent coronal slices, flip angle = 8°, repetition time (TR) = 8.1 ms, echo time (TE) = 4.6 ms, matrix 256 × 256, field of view (FOV) = 176 × 176 × 125 mm^3^. Head immobilization was established using foam pads inside the coil. The acquisition time was 4 min. All the participants were told not to move during the scans.

### VBM-DARTEL Preprocessing

Before VBM-DARTEL preprocessing, all the images were checked for quality. All collected images were checked for artifacts and head movement by two experienced neuroradiologist. Low quality images were eliminated and participants were excluded from further analysis.

All T1-weighted MR image data were processed using Statistical Parametric Mapping 8 (SPM8; Welcome Department of Imaging Neuroscience, London, England^1^) via MATLAB 7.11 (Mathworks, Natick, MA, USA). During the VBM processing, DARTEL was used to improve the inter-participant registration of the structural images. First, the raw data artifacts for each participant were inspected, and each image origin was manually reset so that the millimeter coordinates of the anterior commissure (AC) matched the origin [0, 0, 0], and the orientation approximated the Montreal Neurological Institute (MNI) space at the AC. Next, the T1-weighted MR images were segmented into GM, white matter, and cerebrospinal fluid. After segmentation, we used an initial import routine to generate roughly aligned GM, white matter, and cerebrospinal fluid images for the participants. Subsequently, the structural images for the 60 participants were used to create DARTEL templates, and each voxel was resampled to 1.5 × 1.5 × 1.5 mm^3^. Then, the warped data were smoothed with an 8-mm full width at half maximum (FWHM) and underwent spatial normalization to the MNI space.

### Statistical Analysis

Demographics and clinical variables were assessed using statistical software (SPSS, version 17.0). The group differences in GMV were assessed in VBM-DARTEL using an independent samples *t*-test with whole brain volume, age, gender, education and IQ as covariates. The significance was set at a value of *p* < 0.05 after a family-wise error (FWE) correction for multiple comparisons with a minimum cluster size of 50 voxels.

Voxel of interst (VOI) consisting of the voxels in the regions showing the greatest significant differences (*P*_FWE−corrected_ < 0.05) between the pediatric OCD patients with HCs were defined. These GM areas were selected using the xjView tool in SPM8. The GMVs within these ROIs of patients were extracted using the MarsBar toolbox^2^. Then, the correlation analysis was performed using SPSS between the GMV in these ROIs and clinical variables, which included age, education and the HAM-A, HAM-D, CY-BOCS, and IQ scores.

## Results

### Demographic Data and Clinical Characteristics

A total of 73 participants, including 36 first-episode, treatment-naive pediatric OCD patients and 37 matched HCs were recruited. Due to the poor quality of images and/or heavy head movement, six pediatric OCD patients and seven HCs were excluded from our current study. All the enrolled participant (30 pediatric OCD patients vs. 30 matched HCs) were of the Han Ethnic group. The pediatric OCD patients included 12 girls and 18 boys. The age of onset of pediatric OCD patients was 10.8 ± 2.1 years. Table 1 shows the detailed demographic data and clinical characteristics of the patients and HCs.

**Table 1:** **Demographic data and clinical characteristics**.

Characteristic	OCD group (*n* = 30, *M* = 18)		HC group (*n* = 30, *M* = 18)	
	Mean	SD	Mean	SD
**Age (years)**	10.8	2.1	10.5	2.2
**IQ score**	102.5	7.1	104.4	6.9
**Education (years)**	4.6	2.2	4.5	2.3
**Total CY-BOCS score**	18.3	5.5	2.3	2.8
**Obsessive subscale score**	11.1	3.5	1.6	2.0
**Compulsive subscale score**	10.2	4.1	0.7	0.9
**HAM-A score**	8.4	2.6	3.0	2.8
**HAM-D score**	7.9	2.1	2.6	2.1

*Significant differences in between group comparisons; Abbreviations: IQ, intelligence quotient; CY­BOCS, children’s yale brown obsessive compulsive scale; HCs, healthy controls; HAMA, hamilton anxiety rating scale; HAMD, hamilton depression rating scale; NA, unavailable; M, male.

No significant differences were evident with regard to age, gender, education level and IQ scores between pediatric OCD patients and HCs (Table 1).

### VBM-DARTEL Results

**Figure 1:**
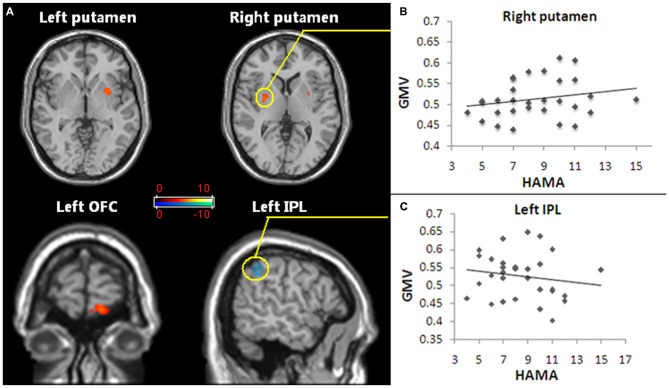
**Regions of gray matter volume (GMV) abnormalities exhibited in pediatric obsessive-compulsive disorder (OCD) patients and the GMV abnormalities correlated with anxiety symptoms. (A)** The red and white voxels reveal the brain areas in which the GMV increased in pediatric OCD, and the blue and green voxels demonstrate reduced GMV regions in pediatric OCD. The regions with increased GMV included the bilateral putamen and the left orbitofrontal cortex (OFC). Reduced GMV included the left inferior parietal lobule (IPL). Statistical significance was assumed with *p* < 0.05 after family-wise error (FWE) correction with a minimum cluster size of 50 voxels (with age, gender and whole brain volume as covariates). **(B)** The increased GMV in the right putamen (the region in the yellow circle) of the pediatric OCD group was positively correlated with HAM-A scores (*p* < 0.05, *r* = 0.44). **(C)** The decreased GMV in the left IPL (the region in the yellow circle) of the OCD group show a trend of was negatively correlated with HAM-A scores (*p* = 0.051, *r* = −0.39). Abbreviations: FWE, family-wise error; GMV, gray matter volume; OCD, obsessive-compulsive disorder; OFC, orbitofrontal cortex; IPL, inferior parietal lobule.

Pediatric OCD patients exhibited increased GMV in the left OFC and bilateral putamen and reduced GMV in left inferior parietal lobule (IPL; Figure 1A; Table 2).

**Table 2:** **Significant gray matter volume (GMV) alterations in pediatric obsessive-compulsive disorder patients vs. healthy controls**.

Brain region	Location	Cluster size (voxels)	*t*-value	Primary peak MNI coordinate (mm)		
**Pediatric**
**OCD > HCs**
**OFC**	Left	76	5.47	−12.0	66	−9
**Putamen**	Left	62	5.21	−27.0	12	0
**Putamen**	Right	54	5.21	31.5	3	45
**Pediatric**
**OCD < HCs**
**IPL**	Left	69	−5.77	−62.0	−51	39

*p*_(FWE-corrected)_ < *0.05 with a minimum cluster size of 50 voxels; Abbreviations: FWE, family-wise error; GMV, gray matter volume; OCD, obsessive-compulsive disorder; HCs, healthy controls, OFC, orbitofrontal cortex; IPL, inferior parietal lobule; MNI, montreal neurological institute*.

### Clinical Correlation

The GMV alteration in the right putamen significantly positively correlated with the HAM-A score (*r* = 0.44, *p* < 0.05; Figure 1B), while the GMV alteration in the left IPL showed a trend to was negatively correlated with the HAM-A scores (*r* = −0.39, *p* = 0.051; Figure 1C).

## Discussion

Compared with HCs, our results revealed increased GMV in the left OFC and bilateral putamen and reduced GMV in the left IPL in first-episode, treatment-naive pediatric OCD patients. The results support our hypothesis that GM abnormalities were defined in the early stage of pediatric OCD and provide further evidence of brain GM abnormalities present not only in the classical fronto–striatal–thalamic circuit but also in the parietal cortex, which might play a supplemental role in the pathophysiology of pediatric OCD. In addition, GMV alterations in the right putamen were found to be positively correlated with HAM-A scores. A trend of negative correlation was also revealed between GMV alterations in the left IPL and HAM-A scores.

Our findings of increased GMV in left OFC and bilateral putamen indicated the malfunction of the frontal-striatal-thalamic circuit. The results are in line with several findings in OCD (Kim et al., 2001; Valente et al., 2005; Szeszko et al., 2008) but contradict some previous reports (Szeszko et al., 1999; Kang et al., 2004; Pujol et al., 2004) in adult OCD and a study in pediatric OCD, which reported a lower OFC volume (Chen et al., 2013). These discrepancies maybe partially explained by the different methodologies, such as voxel-based whole-brain comparison vs. the ROI method, the VBM-DARTEL method vs. the VBM method, and our strict sample enrollment (first-episode and treatment-naïve without any comorbidities).

Sanides (2006) suggested the OFC as part of the “ventral paleo cortical system” in brain development, which might have relevance in the phenomenology of OCD. Dorsal prefrontal brain areas are implicated in the cognitive reappraisal of emotional stimuli, whereas ventral medial prefrontal areas are involved in the execution of reversal learning (Ochsner and Gross, 2005; Mitchell, 2011). In normally developed children, the maturation of OFC precedes that of the dorsal prefrontal cortex (Gogtay et al., 2004; Marsh et al., 2008). The impaired development of OFC could lead to hampered emotional regulation. Therefore, we assume that this ventral-to-dorsal development may be different in pediatric OCD patients and the increased OFC volume might be the result of a compensatory mechanism for the insufficient maturation of the dorsal prefrontal regions.

Neuroimaging studies have indicated OFC as a key element in the fronto–striatal–thalamic circuit of OCD. Anatomically connected with limbic structures (e.g., striatum, hypothalamus) and other prefrontal areas, OFC has been deemed the superior integration center for emotional processing (Gaikwad, 2014). Based on the effects of lesions in humans and animal experiments and on the results of single-unit electrophysiological recordings, the connection of these abovementioned structures has also been implicated in the processes of rewarding or punishing (Neary, 1990). The imbalance of the fronto–striatal–thalamic circuit could represent the neuroanatomic substrate being overwhelmed by internal cues frequently reported by OCD patients, especially when goal-directed behavior is required (Schmidtke et al., 1998), which may be a common neural feature of OCD and OC-spectrum disorders.

The striatum, especially the putamen and caudate nucleus together with their OFC connections, have long been of central interest in OCD (Graybiel and Rauch, 2000; Haruno and Kawato, 2006; Williams and Eskandar, 2006). Baxter et al. (1992) and Rasmussen et al. (2013) proposed that the injury of the striatum could lead to deficiencies in sensory integration with the OFC in OCD. Specifically, the hyperactivity of OFC or lowered striatum filtering together would cause the occurrence of symptoms of OCD.

Hypermetabolism in the putamen of OCD was also reported by a functional neuroimaging study (Perani et al., 1995). Notably, compared to adult OCD, the putamen has more frequently been reported to enlarge than the caudate nucleus in pediatric OCD (Huyser et al., 2009). Considering that the enlargement of the GMV of the right putamen was positively correlated with the anxiety symptoms in our study, the results suggested that the right putamen might be closely associated with anxiety symptoms in pediatric OCD.

Our result of the increased GMV in right putamen of pediatric OCD covered both the dorsal and ventral part of putamen (mainly the dorsal part). And the incresed GMV in left putamen of pediatric OCD mainly covered the dorsal part. These results are consistent with some previous pediatric OCD studies. Gilbert et al. (2008) reported increased GM density of pediatric OCD in the dorsal part of right putamen and Szeszko et al. (2008) reported increase GMV of pediatric OCD in the dorsal putamen. However, these results are partly consistent with previous reports of GMV enlargement of ventral putamen in adult OCD by Pujol et al. (2004) and a meta-analysis by Radua and Mataix-Cols (2009). Pujol et al. (2004) reported increased GMV bilaterally in the ventral putamen and proposed that brain dysfunction of OCD not involve the entire fronto-subcortical system but rather the limbic part such as putamen and OFC. In addition, they identified that age-related volume increased in the ventral putamen of OCD patients and age-related volume decreased in the ventral putamen of HC, similar to what was originally reported in the study by Pujol et al. (2004), de Wit et al. (2014). Therefore, we speculate that adult OCD patients may have larger ventral putamen volume than pediatric OCD patients. The ageing effects and our unique sample characters (first-episode, treatment-naive) may partially explain the discrepancy (dorsal putamen vs. ventral putamen) between our results and those previous reports.

The parietal cortex has been implicated in the modulation of arousal (Heilman, 1997) and negative emotional processing (Etkin and Wager, 2007). Moreover, the parietal cortex has been continuously implicated in the pathophysiology of both adult and pediatric OCD (Pujol et al., 2004; Valente et al., 2005; Carmona et al., 2007). Interestingly, as a part of the suggested default mode network (DMN; Fox et al., 2005), lower activity of IPL has been identified in OCD during simple imagination tasks (Koçak et al., 2011). Stern et al. (2012) chose IPL and several regions of DMN (e.g., posterior cingulate cortex, insular) as seed regions and identified altered functional connectivity in a resting-state between the fronto-parietal network and the DMN, which is considered to be related to OCD symptoms such as the inability to get rid of persisitent intrusive thoughts. Taken together our results that the decreased GMV in the left IPL which exhibited a negative correlation with anxiety symptoms, these findings suggest that anatomic deficits in the DMN regions may share an important function in the genesis or mediation of OCD symptoms.

In summary, our findings of altered GMV in the DMN regions (left IPL) and fronto-striatal regions (OFC, putamen) may represent the interaction of abnormally functional organization of both network.

Given the novelty of the study design, our current study also bears several limitations. First, we did not perform a subgroup analysis with regard to age or gender due to the difficulty in sample enrollment. Further studies with larger sample sizes are warranted to reveal the effects of age or gender that underlie OCD. Second, our results failed to report some frequently discussed brain abnormalities, such as those of the insular and temporal cortices in pediatric OCD. Methodological differences, such as VBM vs. ROI, might partially explain these divergences. Additionally, our study only reported results that reached significance after strict FWE corrections. If the significance of the correction was decreased (e.g., using FDR correction), the results could reveal more divergent regions. Finally, our findings of GMV alterations in pediatric OCD failed to show any correlation with the severity of obsessions and/or compulsions (CY–BOCS scores). This lack of association may have been due to the limited sample size and the small range of CY–BOCS scores, which may not be sufficient to make statistical inferences. Longitudinal studies with large sample sizes to investigate the interaction between different developmental stages of children with OCD are warranted.

To our knowledge, this study is the first to use a 3.0 T MRI system with the VBM-DARTEL method to explore the integrity of whole-brain GM in first-episode, treatment-naive children with OCD. The results of our current study suggest that the GM abnormalities were defined in the early stage of pediatric OCD. In addition, these findings provided further evidence of GM abnormalities in pediatric OCD patients who were present not only in the classical fronto–striatal–thalamic neural circuit but also in the DMN, which may represent the interaction of abnormally functional organization of both network in pediatric OCD. The uniqueness of our pediatric OCD samples (first-episode and treatment-naive without any comorbidities) and study design may contribute to a broader understanding of the etiopathogenesis in OCD.

## Author Contributions

All authors have contributed and have approved the final manuscript. GN took the main responsibility for study design, initiating and writing the manuscript. BC and WC contribute to study design, data collection, data analyses and writing this manuscript. XW, YG and XY were responsible for the data collection and involved in the enrollment of participants. DL, QG, JG and QW contribute to study design, data analysis and editing the manuscript.

## Conflict of Interest Statement

The authors declare that the research was conducted in the absence of any commercial or financial relationships that could be construed as a potential conflict of interest. The reviewer PM and handling Editor declared their shared affiliation, and the handling Editor states that the process nevertheless met the standards of a fair and objective review.

## Footnotes

^1^http://www.fil.ion.ucl.ac.uk/spm/

^2^http://marsbar.sourceforge.net/
